# Newly developed artificial intelligence algorithm for COVID-19 pneumonia: utility of quantitative CT texture analysis for prediction of favipiravir treatment effect

**DOI:** 10.1007/s11604-022-01270-5

**Published:** 2022-04-09

**Authors:** Yoshiharu Ohno, Kota Aoyagi, Kazumasa Arakita, Yohei Doi, Masashi Kondo, Sumi Banno, Kei Kasahara, Taku Ogawa, Hideaki Kato, Ryota Hase, Fumihiro Kashizaki, Koichi Nishi, Tadashi Kamio, Keiko Mitamura, Nobuhiro Ikeda, Atsushi Nakagawa, Yasuko Fujisawa, Akira Taniguchi, Hirotaka Ikeda, Hidekazu Hattori, Kazuhiro Murayama, Hiroshi Toyama

**Affiliations:** 1 Department of Radiology, Fujita Health University School of Medicine, 1-98 Dengakugakubo, Kutsukake-cho, Toyoake, Aichi 470-1192 Japan; 2 Joint Research Laboratory of Advanced Medical Imaging, Fujita Health University School of Medicine, 1-98 Dengakugakubo, Kutsukake-cho, Toyoake, Aichi 470-1192 Japan; 3 Canon Medical Systems Corporation, Otawara, Japan; 4 Departments of Microbiology and Infectious Diseases, Fujita Health University School of Medicine, 1-98 Dengakugakubo, Kutsukake-cho, Toyoake, Aichi 470-1192 Japan; 5 Division of Infectious Diseases, University of Pittsburgh School of Medicine, Pittsburgh, PA USA; 6 Department of Respiratory Medicine, Fujita Health University School of Medicine, 1-98 Dengakugakubo, Kutsukake-cho, Toyoake, Aichi 470-1192 Japan; 7 Center for Clinical Trial and Research Support, Fujita Health University School of Medicine, 1-98 Dengakugakubo, Kutsukake-cho, Toyoake, Aichi 470-1192 Japan; 8 Center for Infectious Diseases, Nara Medical University, Kashihara, Japan; 9 Infection Prevention and Control Department, Yokohama City University Hospital, Yokohama, Japan; 10 Department of Infectious Diseases, Japanese Red Cross Narita Hospital, Narita, Japan; 11 Department of Respiratory Medicine, Isehara Kyodo Hospital, Isehara, Japan; 12 Department of Respiratory Medicine, Ishikawa Prefectural Central Hospital, Kanazawa, Japan; 13 Department of Intensive Care, Shonan Kamakura General Hospital, Kamakura, Japan; 14 Division of Infection Control, Eiju General Hospital, Tokyo, Japan; 15 Department of General Internal Medicine, Eiju General Hospital, Tokyo, Japan; 16 Department of Respiratory Medicine, Kobe City Medical Center General Hospital, Kobe, Japan

**Keywords:** COVID-19, CT, Machine learning, Favipiravir

## Abstract

**Purpose:**

Using CT findings from a prospective, randomized, open-label multicenter trial of favipiravir treatment of COVID-19 patients, the purpose of this study was to compare the utility of machine learning (ML)-based algorithm with that of CT-determined disease severity score and time from disease onset to CT (i.e., time until CT) in this setting.

**Materials and methods:**

From March to May 2020, 32 COVID-19 patients underwent initial chest CT before enrollment were evaluated in this study. Eighteen patients were randomized to start favipiravir on day 1 (early treatment group), and 14 patients on day 6 of study participation (late treatment group). In this study, percentages of ground-glass opacity (GGO), reticulation, consolidation, emphysema, honeycomb, and nodular lesion volumes were calculated as quantitative indexes by means of the software, while CT-determined disease severity was also visually scored. Next, univariate and stepwise regression analyses were performed to determine relationships between quantitative indexes and time until CT. Moreover, patient outcomes determined as viral clearance in the first 6 days and duration of fever were compared for those who started therapy within 4, 5, or 6 days as time until CT and those who started later by means of the Kaplan–Meier method followed by Wilcoxon’s signed-rank test.

**Results:**

% GGO and % consolidation showed significant correlations with time until CT (*p* < 0.05), and stepwise regression analyses identified both indexes as significant descriptors for time until CT (*p* < 0.05). When divided all patients between time until CT of 4 days and that of more than 4 days, accuracy of the combined quantitative method (87.5%) was significantly higher than that of the CT disease severity score (62.5%, *p* = 0.008).

**Conclusion:**

ML-based CT texture analysis is equally or more useful for predicting time until CT for favipiravir treatment on COVID-19 patients than CT disease severity score.

## Introduction

The new coronavirus disease 2019 (COVID-19) has been spreading worldwide since late 2019 and become a global pandemic involving over 200 countries or regions and more than 180 million individuals. About 10–20% of COVID-19 patients deteriorate into severe or critical illnesses within 7–14 days after symptom onset. This deterioration is characterized by acute respiratory distress syndrome (ARDS) or even multiorgan dysfunction syndrome (MODS), thus requiring more intensive medical resource utilization with a tendency to develop nosocomial complications, which lead to a worse prognosis with a case fatality rate about 20 times higher than that for non-severe patients [1–3].

Recent advances in artificial intelligence (AI) and specifically in machine learning (ML) have led to substantial changes in medical imaging. AI software based on various ML-based approaches for thoracic images such as chest radiography and computed tomography (CT) have already contributed to the fight against the COVID-19 pandemic, particularly in assisting the diagnosis, stratification, prognosis, and treatment of COVID-19 patients, but only several approaches have been validated by radiotherapists’ findings [4].

In contrast to the role of radiology in the overall management of COVID-19, there is no specific anti-coronavirus treatment for severe patients at present, and whether the antiviral agent remdesivir is associated with significant clinical benefits for severe COVID-19 still requires further confirmation [5, 6]. Favipiravir, which is an oral, broad-spectrum inhibitor of viral RNA-dependent RNA polymerase [7, 8], is currently approved in Japan for the treatment of emerging and reemerging influenza virus infection for which other anti-influenza drugs are ineffective or not sufficiently effective [9]. Favipiravir has demonstrated in vitro activity against SARS-CoV-2, and several randomized studies of COVID-19 conducted in China, Russia, and India have indicated the potential clinical benefit of favipiravir such as shorter time until viral clearance among patients with mild-to-moderate COVID-19 patients, higher rate of viral clearance on the fifth day among hospitalized COVID-19 patients, and shorter time to clinical cure for mild-to-moderate COVID-19 patients, when compared with standard of care [10–12]. A randomized trial of patients with asymptomatic to mildly symptomatic COVID-19 was also conducted in Japan, and although it did not produce significantly improved viral clearance during the first 6 days of treatment, favipiravir was found to be associated with numerical reduction in time to defervescence, and a significant improvement in fever observed the day after starting therapy, suggesting that it has potential for modest clinical benefits. Radiological severity was not considered in that study. For the current study, we developed a new ML-based CT texture analysis software for COVID-19, which evaluates radiological findings in lieu of expert chest radiologists and also functions as a second reader of CT images for various pulmonary diseases [13]. However, it has not been evaluated in terms of predicting therapeutic outcomes for COVID-19 patients. The purpose of this study was to determine the utility of the algorithm for predicting the therapeutic effect of favipiravir therapy for patients who participated in a randomized trial with reference to qualitatively assessed disease severity on CT and time from disease onset to CT.

## Materials and methods

### Protocol, support, and funding

This study was a retrospective study and approved by the institutional review board of Fujita Health University Hospital with written informed consent waved for this particular sub study. This study was financially and technically supported by the Japan Agency for Medical Research and Development (AMED) (JP19fk0108150 and JP20fk0108150), the Grants-in-Aid for Scientific Research from the Japanese Ministry of Education, Culture, Sports, Science and Technology (JSTS.KAKEN; No. 18K07675 and JSTS.KAKEN; No. 20K08037), Smoking Research Foundation and Canon Medical Systems Corporation. Four of the authors are employees of Canon Medical Systems (K.A., Ka. Ara., Y.F. and A.T.) who did not have control over any of the data used in this study.

### Subjects

This was a retrospective analysis of imaging data of subjects who had been included in an investigator-initiated, individually randomized, open-label trial to assess the efficacy and safety of oral favipiravir for adolescents and adults (aged ≥ 16 years) admitted to hospital with asymptomatic to mildly symptomatic COVID-19 [14]. The study was centrally approved by the certified review board of [Blinded], which served as the coordinating center, and subsequently approved by the director of each participating hospital prior to site initiation. Written informed consent was obtained from all study participants for the trial.

**Fig. 1:**
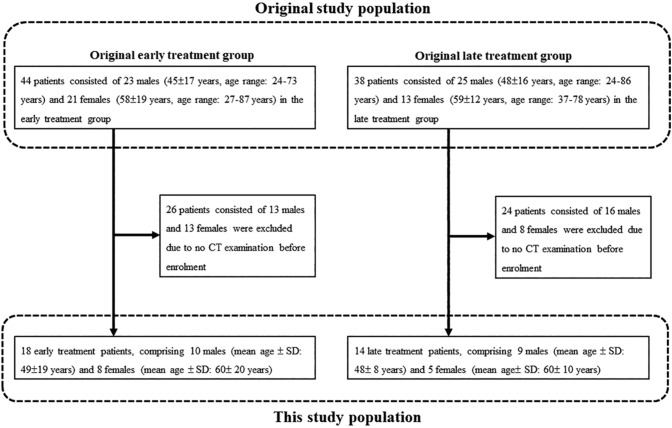
Patients’ flowchart

From 2 March to 18 May 2020, original patients were recruited at 25 hospitals across Japan, and the follow-up was completed on 14 June 2020. The inclusion criteria for the trial were: (1) age 16 years or older, (2) inpatient status, (3) positive reverse transcription polymerase chain reaction (RT-PCR) for SARS-CoV-2 from a pharyngeal or nasopharyngeal swab specimen collected within 14 days, (4) Eastern Cooperative Oncology Group (ECOG) performance status of 0 or 1 [15], (5) ability to remain hospitalized for 6 days or longer, (6) negative pregnancy test (premenopausal females only), and (7) written consent for participation. The exclusion criteria were: (1) performance status 2 or higher, (2) severe hepatic disease, (3) need for dialysis, (4) altered mental status, (5) pregnancy, (6) female patients who refused to use effective contraceptive methods, (7) male patients with female partners who refused to use effective contraceptive methods, (8) hereditary xanthinuria, (9) hypouricemia or history of xanthine urolithiasis, (10) uncontrolled gout or hyperuricemia, (11) immunosuppressive conditions, and (12) receipt of systemic antiviral agent against SARS-CoV-2 within preceding 28 days. A total of 89 patients (mean age ± SD: 52 ± 18 years) with laboratory-confirmed COVID-19 were randomized: 44 were assigned to the early treatment group and 45 to the late treatment group. One subject withdrew consent immediately after consenting to the study, leaving 88 patients consisting of 54 males (mean age ± SD: 47 ± 16 years, age range: 24–86 years) and 34 females (mean age ± SD: 59 ± 17 years, age range: 27–87 years) with any of their study-related data and included the intention-to-treat (ITT) population. The infected ITT population, so defined for the primary outcome analysis of viral clearance, consisted of 36 and 33 patients in the early and late treatment groups after the exclusion of 8 and 11 patients in the respective groups whose RT-PCR result on the first day was already negative. The safety population included 44 patients consisting of 23 males (45 ± 17 years, age range: 24–73 years) and 21 females (58 ± 19 years, age range: 27–87 years) in the early treatment group and 38 patients consisting of 25 males (48 ± 16 years, age range: 24–86 years) and 13 females (59 ± 12 years, age range: 37–78 years) in the late treatment group after the exclusion of seven patients who did not receive any favipiravir dose. The day of randomization was day 1 for 86 patients. For the remaining three patients (two in the early and one in the late treatment groups), day 1 was the day following randomization since randomization took place too late in the evening for the two first-day doses to be given if assigned to the early treatment group. Details of randomization and procedures have been reported in the past literature [14]. The two groups were similar in their overall demographic and clinical characteristics as well as baseline laboratory results, but there was an imbalance in the male-to-female ratio, with males accounting for 52.3% in the early treatment group and 70.5% in the late treatment group [14]. All participants in early treatment group were started on favipiravir on day 1 (early treatment group), and those in late treatment group were started on it on day 6 (late treatment group). Among the 44 and 38 patients in the early and late treatment groups, CT images prior to enrollment were available for 32 patients (mean age ± SD:51 ± 18 years) consisting of 18 patients in the early treatment group (ten males [mean age ± SD: 49 ± 19 years] and eight females [mean age ± SD: 60 ± 20 years]) and 14 patients in the late treatment group (nine males [mean age ± SD: 48 ± 8 years] and five females [mean age ± SD: 60 ± 10 years]). In addition, time between onset of clinical symptoms and CT examination was also recorded. Flowchart for patient selection is shown in Fig. 1, and details of patients’ characteristics are shown in Table 1.

**Table 1:** Demographic and clinical data, quantitative radiological indexes, and qualitative CT severity scores for early and late treatment groups

		Early treatment (*n* = 18)	Late treatment (*n* = 14)	*p* value
Age (years)	(Mean ± standard deviation)	51 ± 19	52 ± 12	0.43
Gender	Male: female	10:8	9:5	0.32
Height (cm)	(Mean ± standard deviation)	163.4 ± 10.5	165.1 ± 9.2	0.59
Body weight (Kg)	(Mean ± standard deviation)	62.2 ± 16.1	67.7 ± 10.3	0.17
Clinical symptoms	Number (%)	18 (100)	12 (85.7)	0.07
Fever	Number (%)	10 (55.6)	8 (57.1)	0.93
Time between onset of clinical symptoms and CT examination (days)	(Mean ± standard deviation)	5.8 ± 3.3	5.9 ± 3.7	0.88
Quantitative indexes (%: mean ± standard deviation)	Normal lung	86.6 ± 5.6	86.9 ± 7.9	0.56
	GGO	7.1 ± 4.7	7.1 ± 4.8	0.99
	Reticulation or crazy paving	3.7 ± 3.8	4.2 ± 4.7	0.81
	Emphysema	0.7 ± 1.7	0.5 ± 1.1	0.59
	Nodular lesion	0.0 ± 0.0	0.0 ± 0.0	0.27
	Consolidation	1.8 ± 2.3	1.2 ± 1.8	0.47
	Honeycombing	0.1 ± 0.1	0.1 ± 0.1	0.66
CT disease severity score	(Mean ± standard deviation)	5.6 ± 4.7	7.1 ± 3.9	0.24

*GGO* ground-glass opacity

### CT examinations

The CT data were obtained with ten 64- and three 256-detector row CT scanners (Optima 660 Pro and Revolution; GE Healthcare, Milwaukee, WI), three 80- and three 320-detector row CT scanners (Aquilion PRIME and Aquilion ONE; Canon Medical Systems, Otawara, Tochigi, Japan), or four 64-detector row CT scanners (SOMATOM Sensation Cardiac 64 and Definisition AS + , Siemens Healthineers, Erlangen, Germany). CT examinations were performed as unenhanced CT with helical scanning using the following parameters: 64–80 × 0.5–0.624 mm collimation, auto mA, 120kVp, 0.55–1.35 beam pitch, 0.5 s gantry rotation time, 512 × 512 matrix and 280–370 mm field of view. All thin-section CT data were then reconstructed with the filtered back projection provided by all vendors or hybrid iterative reconstruction methods such as AIDR 3D (Canon Medical) or ASiR (GE Healthcare) in contiguous section thicknesses of 1 mm and used for generating the standard reconstruction kernel provided by each vendor. The estimated volume computed tomography dose index (CTDI_vol_ [e.g., and the following parameters]) displayed on the CT scanner console was recorded for each patient. These values were based on the weighted computed tomography dose index (CTDI_w_ [e.g., tube voltage or tube current]). CTDI_vol_ obtained in this study was assessed as 10.6 ± 5.6 (mean ± SD) mGy and ranged between 3.4 and 24.2 mGy. The estimated dose–length product (DLP) was calculated as CTDI_vol_ × scan length, which was determined as 121.4–682.9 mGy × cm, with the effective dose for this protocol estimated at 1.7–9.5 mSv. All CT examinations were performed with breath holding at full inspiration.

### Treatment procedures and patient outcomes

Favipiravir was dosed at 1,800 mg twice orally at least 4 h apart on the first day, followed by 800 mg orally twice a day, for a total of up to 19 doses over 10 days. This regimen achieves plasma concentration of approximately 60 μg/ml and higher in healthy individuals (data on file, FUJIFILM Toyama Chemical, Tokyo, Japan). If the patients met the discharge criteria sanctioned by the government (resolution of symptoms and two serial negative RT-PCR test results performed locally) during the study period, and they had reached at least the sixth day of study participation, they were allowed to discontinue favipiravir, discharged home, and followed up at the end of the study either in person or by phone. Use of other medications with antiviral activity was prohibited during the course of study participation. Nasopharyngeal swabs were collected daily between day 1 and day 6 and then every other day through day 16 if the patient remained in hospital. RT-PCR was conducted at a centralized study laboratory using the protocol that was developed at the National Institute of Infectious Diseases and widely adopted in Japan [16]. Details have been specified in the literature [14]. In accordance with the multicenter study design and results [14], viral clearance in the first 6 days, duration of fever (≥ 37.5 °C or ≥ 37.0 °C), and time until hospital discharge were recorded as patient outcomes in this study.

### Image analysis

#### Quantitative radiological finding evaluation by machine learning software

To quantitatively evaluate the radiological findings as well as disease severity on CT, all measurements by means of the newly developed ML-based CT texture analysis software were performed by a board certified radiologist (Y.O.) with 28 years of experience using a commercially available workstation (Vitrea; Vital Images, Inc., Minnetonka, MN). The software used in this study was proprietary (CT Lung Parenchyma Analysis, Prototype ver. 4) and was provided by Canon Medical Systems and installed on the same workstation. Basics of the three-dimensional (3D) ML-based texture analysis software was described in the past literature [13, 17], and this section is briefly mentioned the algorithm.

**Fig. 2:**
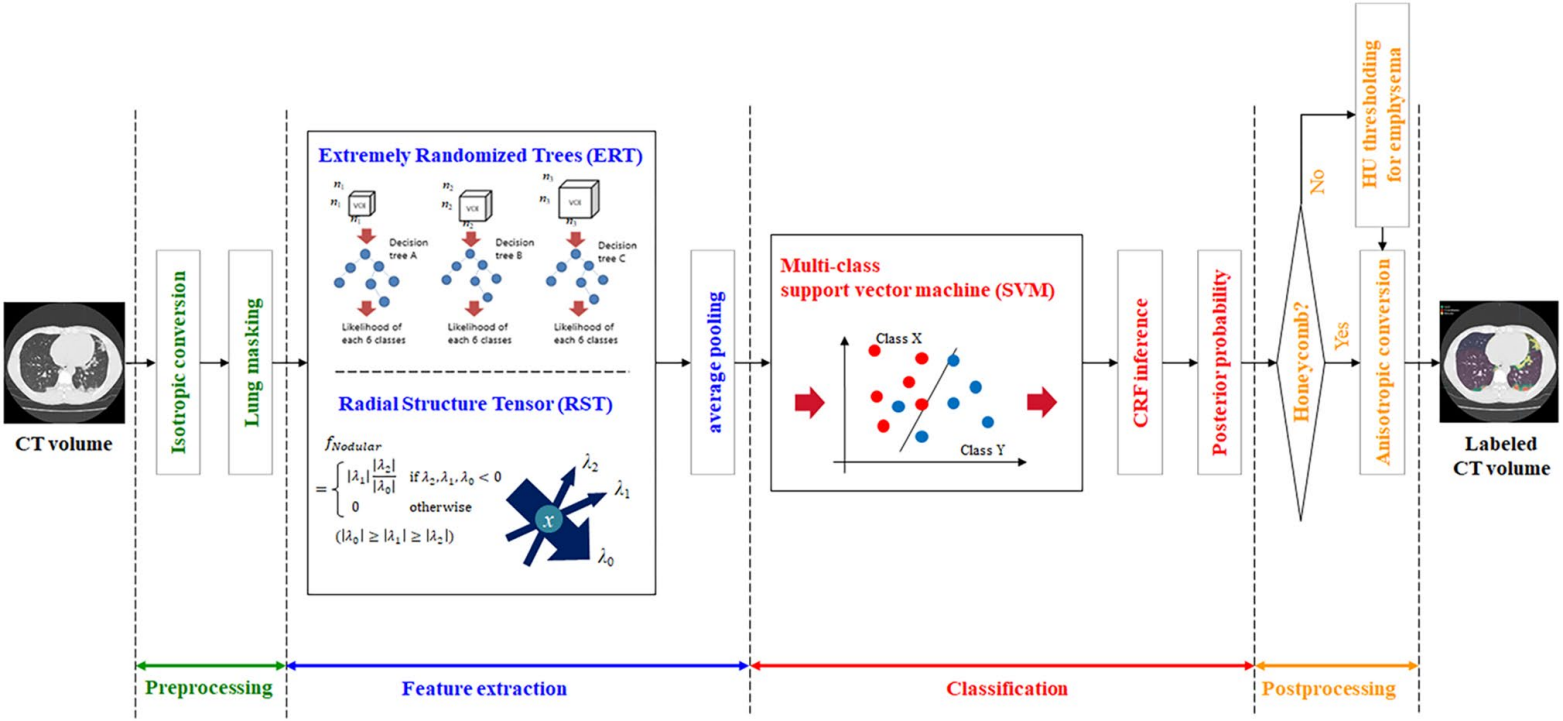
Flowchart of three-dimensional (3D) machine learning for CT texture analysis. Flowchart of the proposed method. At the feature extraction stage, likelihood of each texture pattern’s occurrence on every voxel is calculated. At the classification stage, probability of each texture pattern is calculated from the features extracted on each voxel. Finally, each voxel is labeled with a specific texture pattern showing the maximum posterior probability

Figure 2 shows a schematic diagram of the ML-based texture analysis algorithm in this study. The algorithm is designed to classify every single voxel into seven radiological finding-based categories derived from the glossary terms for thoracic imaging published by the Fleischner Society [18]: (1) normal lung, (2) ground-glass opacity (GGO), (3) reticulation, (4) emphysema, (5) nodular lesion, (6) consolidation, and (7) honeycomb.

Given a set of chest CT images as input, it is converted to an isotropic volume with 0.6 mm spacing and the lung region is extracted as the preprocessing. In the feature extraction stage, the feature vector of each voxel is calculated by means of the extremely randomized trees (ERT) [19] and the radial structure tensor (RST) [20]. ERT is a tree-based ensemble method for supervised classification and is trained to infer voxel-wise likelihoods of six texture categories excluding nodular lesion in multiple scale. RST is a filter that enhances blob-like structures by correlating position and direction of the gradient vectors in a local neighborhood and is utilized for extracting likelihood of nodular lesion. Then, we apply average pooling with multiple local window sizes to the extracted feature vectors. In the classification stage, the voxel-wise probability of each texture category is calculated from the extracted features using the multiclass support vector machine (SVM) [21], which is a set of supervised learning methods used for classification. Then, the output probabilities of SVM are corrected using conditional random field (CRF) [22] to provide optimal probabilities for a whole volume by considering differences in both location and voxel values between adjacent voxels. Finally, each voxel is labeled with a specific texture category with the maximum posterior probability. The voxels with a Hounsfield unit (HU) below − 950 are relabeled with emphysema. Note that the voxels with a honeycomb label are excluded for this simple thresholding as this texture category may contain voxels below − 950 HU.

Each lesion volume, normalized by the lung volume determined from CT data, was then automatically calculated, while all radiologically determined volumes (% normal lung, % emphysema, % nodular lesion, % consolidation, % GGO, % reticulation, and % honeycombing) were determined as a percentage of total lung volume.

#### Qualitative CT severity score assessment based on previously published scoring system

To evaluate the disease severity of COVID-19, qualitatively assessed disease severity was independently scored by two chest radiologists ([Blinded] and [Blinded]) with 17 and 28 years of experience with the same workstation, respectively. Both reviewers assessed disease severity without having access to any information about clinical symptoms, RT-PCR data, or treatment group assignment for any patient. Then, the final qualitative CT severity score was determined as the averaged value from two investigators in each patient. For all cases, a qualitative CT severity scoring method proposed by Pan et al. [23] was used to calculate the extent of anatomic involvement for each of the 5 lobes, as follows: 0, no involvement; 1, < 5% involvement; 2, 5–25% involvement; 3, 26–50% involvement; 4, 51–75% involvement; and 5, > 75% involvement. The resultant global CT score was calculated by summing the individual lobar scores, with a possible range of a minimum of 0 to a maximum of 25.

### Statistical analysis

To compare early and late treatment groups in this study, gender, age, clinical symptoms, and time between onset of clinical symptoms and CT examination (i.e., time until CT) were compared using Chi-square test, Wilcoxon’s signed-rank test, or Student’s t test.

The relationships between quantitative and qualitative radiological indexes and time until CT were determined by means of univariate and stepwise regression analyses. 

To determine feasible threshold values for each quantitative index, combined quantitative method and qualitative index, receiver-operating characteristics (ROC)-based positive tests were performed to differentiate patients whose time until CT was equal to or less than 4 days from those whose time until CT was more than 4 days, and similarly for those whose time until CT was equal to or less than and more than 5 or 6 days, respectively. Sensitivity, specificity, and accuracy for differentiation of patients whose time until CT was equal to or less than 4, 5, or 6 days from those whose time until CT was more than the corresponding number of days were determined for all comparisons by means of McNemar’s test. Sensitivity, specificity, positive predictive value, negative predictive value, and accuracy were calculated for each level of these indexes by varying the levels of indexes that signified a positive test (threshold value) [24–26]. To differentiate patients whose time until CT was equal to or less than 4, 5, or 6 days from those whose time until CT was more than the corresponding number of days, sensitivity was defined as the percentage of patients whose time until CT was equal to or less than 4, 5, or 6 days whose level of indexes was above the given threshold level. Specificity was defined as the percentage of patients whose time until CT was more than 4, 5, or 6 days whose level of indexes was less than or equal to the threshold levels.

To compare outcomes for each patient enrolled in this study, Kaplan–Meier analysis followed by Wilcoxon’s signed-rank test was performed to compare patients whose time until CT was equal to or less than 4, 5, or 6 days in early treatment group and those whose time CT was more than the corresponding number of days for all radiological indexes as well as time until CT in early treatment group with all patients in late treatment group.

## Results

**Fig. 3:**
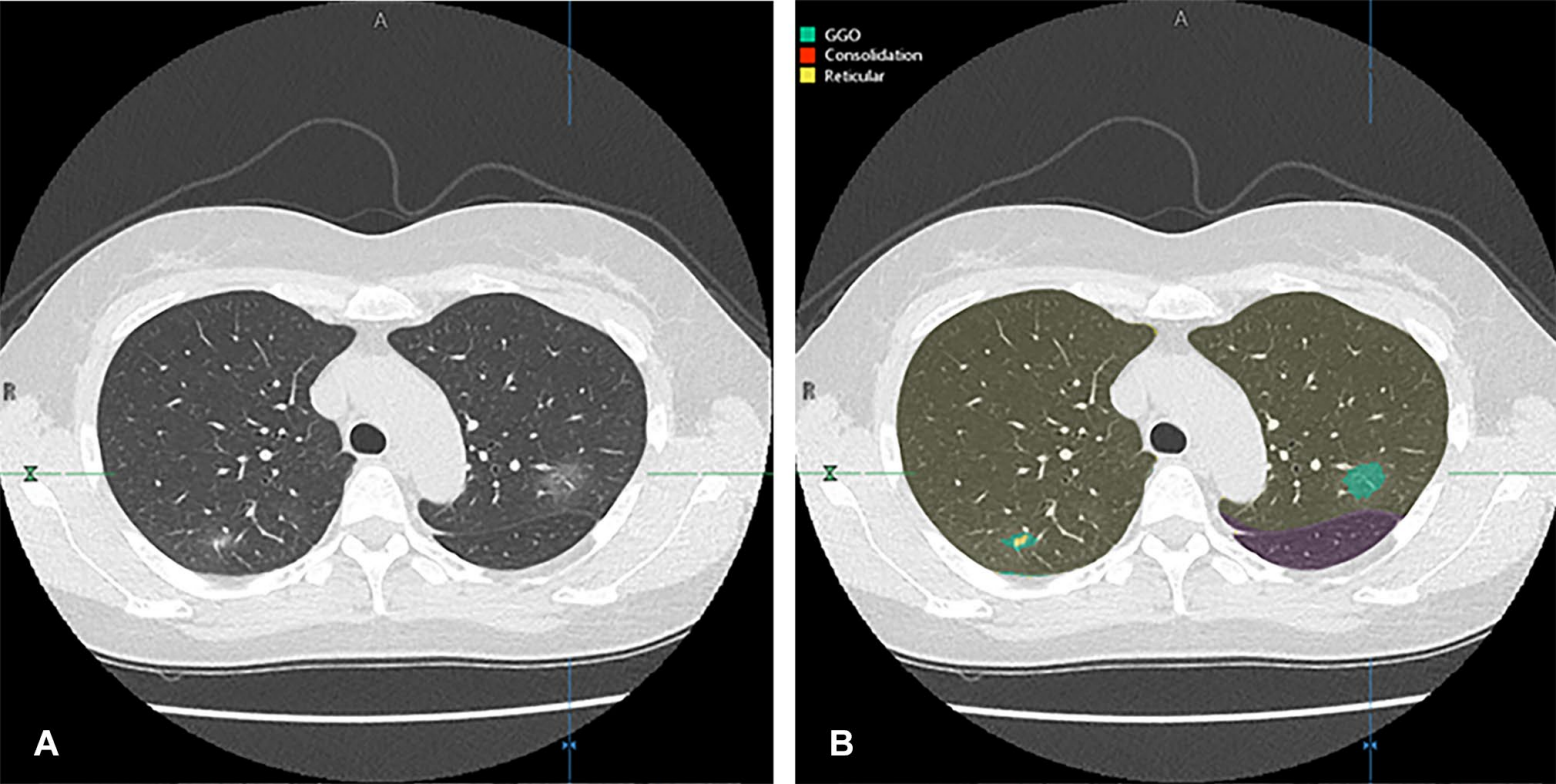
A 53-year-old female COVID-19 patient whose CT image was obtained 3 days after onset of clinical symptoms and assigned to the early treatment group in the original multicenter study. **A** Thin-section CT shows ground-glass opacities (GGOs) in the bilateral upper lobes. **B** Thin-section CT analyzed using the machine learning-based software shows GGOs as green areas and reticulation as a yellow area. % GGO in this case was assessed as 6%, and % consolidation as 0.3%. Probability within 4, 5, and 6 days from clinical onset determined with the combined method as 0.58, 0.58, and 0.64, respectively. CT disease severity score was 3. Prediction for this patient assigned to the early treatment group was based on %GGO, % consolidation, combined method, and CT disease severity score. After administration of favipiravir, periods for viral clearance, duration of fever, and time until hospital discharge were 1 day, 1 day, and 14 days, respectively

**Fig. 4:**
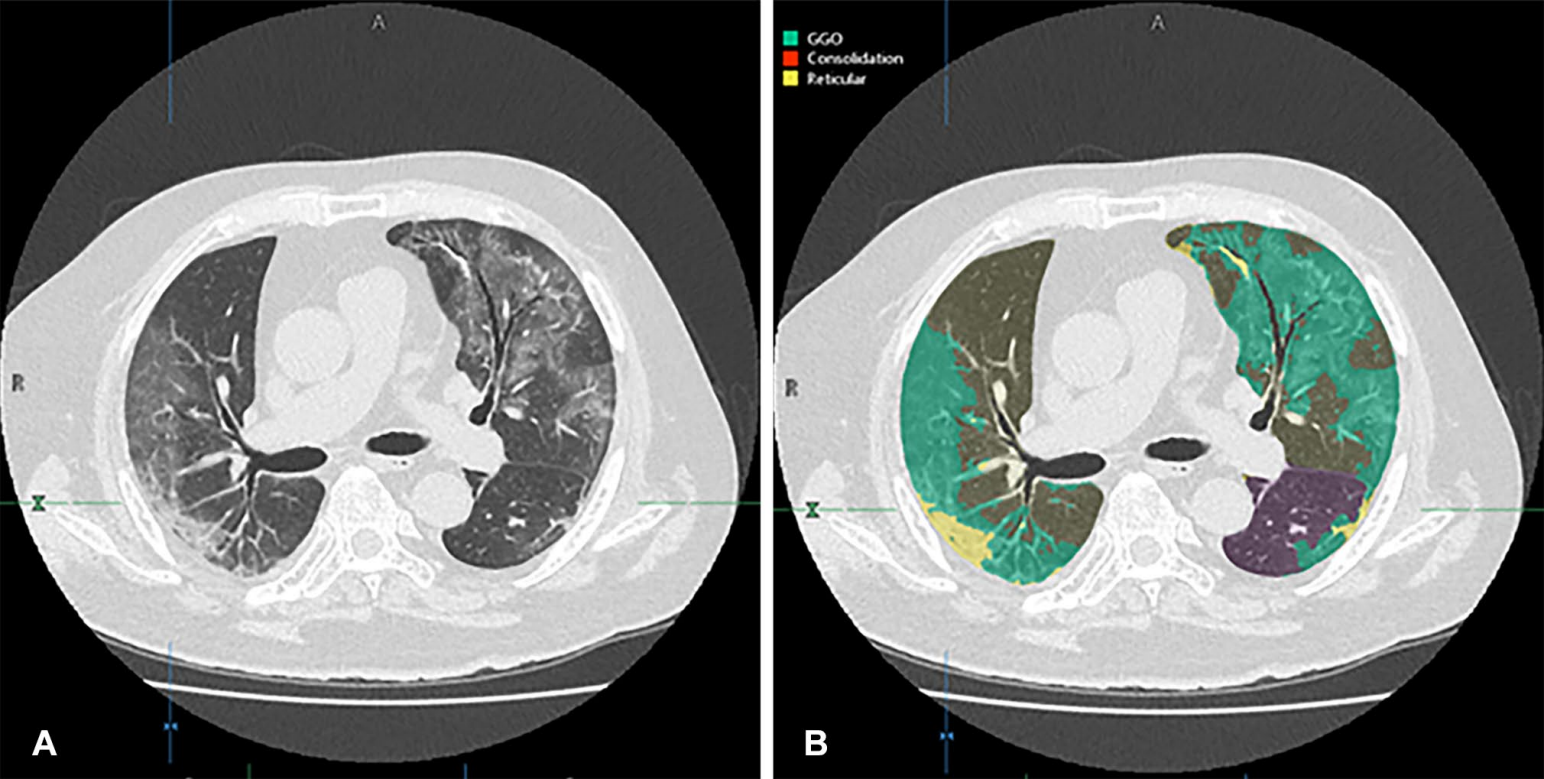
A 27-year-old male COVID-19 patient whose CT image was obtained 7 days after onset of clinical symptoms and assigned to the early treatment group in the original multicenter study. **A** Thin-section CT shows ground-glass opacities (GGOs) and reticulations in the bilateral lungs. **B** Thin-section CT analyzed using the machine learning-based software shows GGOs as green areas and reticulation as a yellow area. % GGO in this case was assessed as 16.8%, and % consolidation as 2.8%. Probability within 4, 5, and 6 days from clinical onset determined with the combined method as 0.62, 0.56, and 0.74, respectively. CT disease severity score was 17. Prediction for this patient assigned to the early treatment group was based on % consolidation and combined method. After administration of favipiravir, periods for viral clearance, duration of fever, and time until hospital discharge were 2 days, 1 day, and 14 days, respectively

**Fig. 5:**
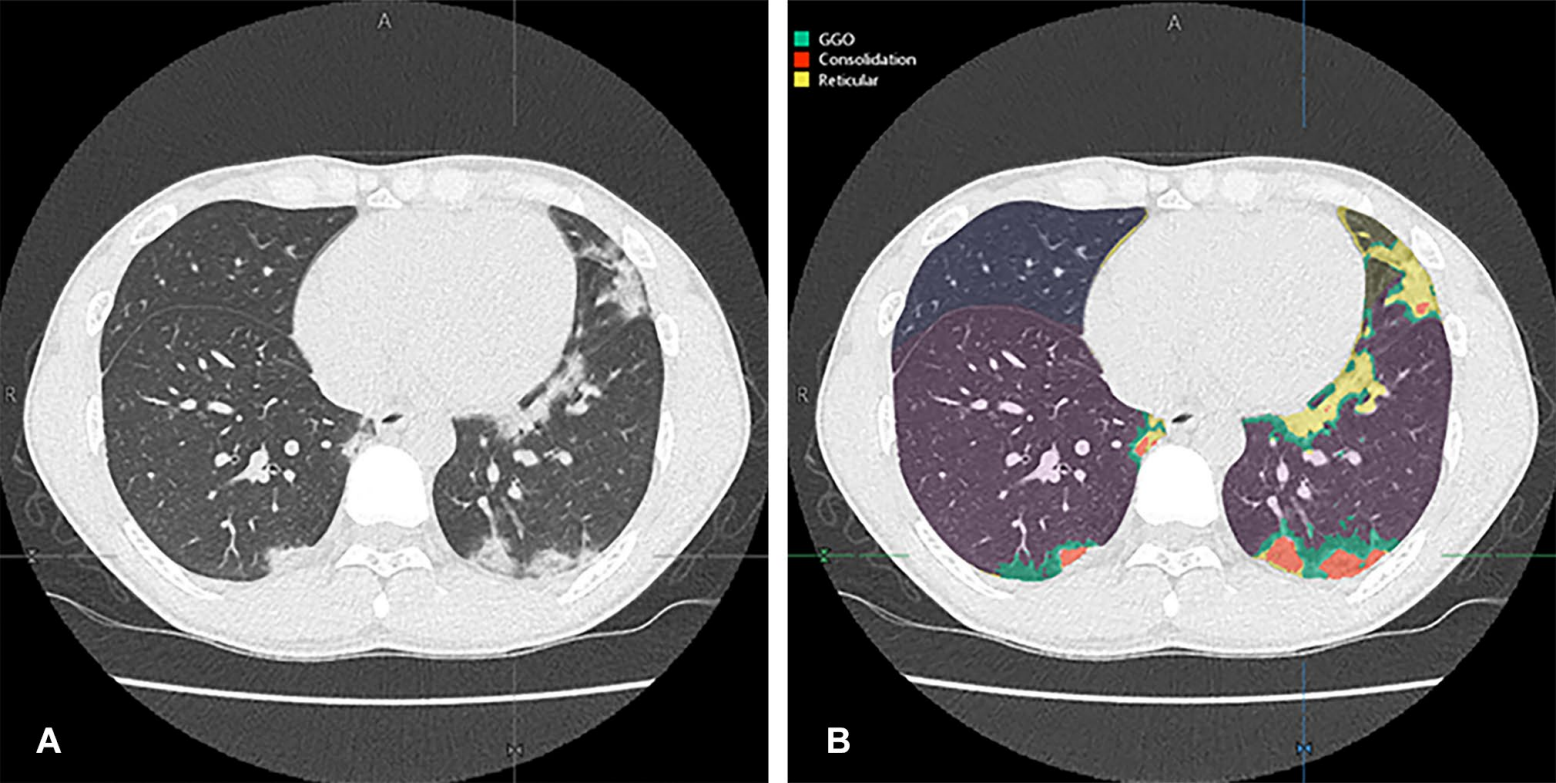
A 29-year-old female COVID-19 patient whose CT image was obtained 10 days after onset of clinical symptoms and assigned to the early treatment group in the original multicenter study. **A** Thin-section CT shows CGOs, reticulation and consolidation in both lungs. **B** Thin-section CT analyzed using the machine learning-based software shows GGOs as green areas, reticulation as a yellow area and consolidation as an orange area. % GGO in this case was assessed as 2.5%, and % consolidation as 8.9%. Probability within 4, 5, and 6 days from clinical onset determined with the combined method as 0.23, 0.23, and 0.31, respectively. CT disease severity score was 5. Prediction for this patient assigned to the early treatment group was based on only %GGO, and others were accurately predicted as late response case. After administration of favipiravir, periods for viral clearance, duration of fever and time until hospital discharge were 5 days, 3 days, and 21 days, respectively

The demographic and clinical data, quantitative radiological indexes, and qualitative CT severity scores for early and late treatment groups are shown in Table 1. There were no significant differences in any of the demographic and clinical data and quantitative and qualitative indexes between the two groups (*p* > 0.05). Representative cases are shown in Figs. 3, 4, and 5.

Results of correlation between time until CT and all radiological indexes at the initial CT examination are shown in Table 2. Time until CT examination correlated significantly with % GGO (*r* = − 0.45, *p* = 0.005), % consolidation (*r* = 0.48, *p* = 0.002), and CT disease severity score (*r* = 0.45, *p* = 0.008).

**Table 2:** Correlations between time until CT and all radiological indexes at initial CT examination

		Correlation coefficient (*r*)	*p* value
Quantitative index	% Normal lung	0.08	0.64
	% GGO	− 0.45	0.005
	% Reticulation	0.12	0.47
	% Consolidation	0.48	0.002
	% Emphysema	0.06	0.71
	% Nodular lesion	− 0.13	0.45
	% Honeycombing	− 0.24	0.15
Qualitative index	CT disease severity score	0.46	0.009

Stepwise regression analysis between time until CT and all radiological indexes on initial CT examination showed that time until CT was significantly affected by two factors, % consolidation as the first step and % GGO as the second step (r^2^ = 0.31, *p* = 0.03).

Results for differentiating patients whose time until CT was equal to or less than 4, 5, or 6 days from those whose time until CT was more than the corresponding number of days are shown in Tables 3, 4 and 5. When each threshold value was used for time until CT of 4 days, accuracy of the combined quantitative method (87.5% [28/32]) was significantly higher than that of the CT disease severity score (62.5% [20/32], *p* = 0.008). Moreover, sensitivities of % consolidation (94.4% [17/18]) and the combined method (94.4% [17/18]) were significantly higher than that of % GGO (55.6% [10/18], *p* < 0.05), when each feasible threshold value was for time until CT of 5 days.

**Table 3:** Differences between patients whose time until CT was 4 days or less and those whose time until CT was more than 4 days

			TV	SE (%)	SP (%)	PPV (%)	NPV (%)	AC (%)	Real early and late treatment groups (cases)	Viral clearance in the first 6 days (days) [median]	Duration of fever (≥ 37.5 °C or ≥ 37.0 °C) (days) [median]	Time duration until hospital discharge (days) [median]
Radiological prediction	% GGO	4 days or less	10.0 ≤	69.2 (9/13)	89.5 (17/19)	81.8 (9/11)	81.0 (17/21)	81.3 (26/32)	6	1	1	13
		More than 4 days							26	5	3.5	23
		*p* value	N/A						N/A	< 0.0001	< 0.0001	< 0.0001
	% Consolidation	4 days or less	≤ 2.0	100.0 (13/13)	52.6 (10/19)	59.1 (13/22)	100.0 (10/10)	71.9 (23/32)	12	4	2	16
		More than 4 days							20	5	3.5	23.5
		*p* value	N/A						N/A	0.01	0.01	0.008
	Combined method	4 days or less	0.45 <	92.3 (12/13)	84.2 (16/19)	80.0 (12/15)	94.1 (16/17)	87.5 (28/32)	10	3.5	1.5	16
		More than 4 days							22	5	4	23
		*p* value	N/A						N/A	0.007	< 0.0001	0.02
	CT disease severity score	4 days or less	≤ 4	61.5 (8/13)	63.2 (12/19)	57.1 (8/14)	70.6 (12/17)	62.5 (20/32)	7	3	1.5	18
		More than 4 days							25	5	3.5	24
		*p* value	N/A					GGO: *p* = 0.03, Combined method: *p* = 0.008	N/A	0.04	0.001	0.12
Patients whose time until CT equal to or less than 4 days in early treatment group vs. patients assessed as time until CT more than 4 days in early treatment group and all patients in late treatment group		4 days or less	N/A						6	3.5	1.5	15
		More than 4 days							26	5	3.5	23
		*p* value							N/A	0.04	0.0007	0.004

*TV* threshold value, *SE* sensitivity, *SP* specificity, *PPV* positive predictive value, *NPV* negative predictive value, *AC* accuracy

**Table 4:** Differences between patients whose time until CT was 5 days or less and those whose time until CT was more than 5 days

			TV	SE (%)	SP (%)	PPV (%)	NPV (%)	AC (%)	Real early and late treatment groups (cases)	Viral clearance in the first 6 days (days) [median]	Duration of fever (≥ 37.5 °C or ≥ 37.0 °C) (days) [median]	Time duration until hospital discharge (days) [median]
Radiological prediction	% GGO	5 days or less	10.0 ≤	55.6 (10/18)	92.9 (13/14)	90.9 (10/11)	61.9 (13/21)	71.9 (23/32)	6	1	1	13
		More than 5 days							26	5	4	23
		*p* value	N/A	% Consolidation: *p* = 0.02, Combined method: *p* = 0.02	N/A				N/A	< 0.0001	< 0.0001	< 0.0001
	% Consolidation	5 days or less	≤ 2.0	94.4 (17/18)	57.1 (8/14)	73.9 (17/23)	88.9 (8/9)	78.1 (25/32)	12	4	2	16
		More than 5 days							20	5	3.5	23.5
		*p* value	N/A						N/A	0.01	0.02	0.008
	Combined method	5 days or less	0.40 <	94.4 (17/18)	57.1 (8/14)	73.9 (17/23)	88.9 (8/9)	78.1 (25/32)	11	4	2	16
		More than 5 days							21	5	4	23
		*p* value	N/A						N/A	0.02	0.0003	0.008
	CT disease severity score	5 days or less	≤ 4	72.2 (13/18)	85.7 (12/14)	86.7 (13/15)	70.6 (12/17)	78.1 (25/32)	7	3	2	15
		More than 5 days							25	5	3.5	23
		*p* value	N/A						N/A	0.04	0.01	0.01
Patients whose time until CT equal to or less than 5 days in early treatment group vs. patients assessed as time until CT more than 5 days in early treatment group and all patients in late treatment group		5 days or less	N/A						8	4	2	15.5
		More than 5 days							24	5	3	23
		*p* value							N/A	0.05	0.11	0.002

*TV* threshold value, *SE* sensitivity, *SP* specificity, *PPV* positive predictive value, *NPV* negative predictive value, *AC* accuracy

The patient outcome differences for each method and time until CT using days 4, 5, or 6 as cutoffs are also shown in Tables 3, 4, and 5. When differentiated patients whose time until CT was equal to or less than 4 days from more than 4 days by each radiological method and real time until CT in early treatment group, all clinical outcomes showed significant differences between patients assessed as time until CT equal to or less than 4 days in early treatment group and those assessed as time until CT more than 4 days in early treatment group and all patients in late treatment group (*p* < 0.05). When differentiated patients whose time until CT was equal to or less than 5 days from more than 5 days by each radiological method and real time until CT, each patient outcome demonstrated significant difference between patients assessed as time until CT equal to or less than 5 days in early treatment group and those assessed as time until CT more than 5 days in early treatment group and all patients in late treatment group (*p* < 0.05). However, hospital discharge of patient divided by real time until CT as equal to or less 5 days in early treatment group had significant difference with that as more than 5 days in early treatment group and all patients in late treatment group (*p* < 0.05). When differentiated patients whose time until CT was equal to or less than 6 days by combined method, each patient outcome showed significant difference between patients assessed as time until CT equal to or less than 6 days in early treatment group and those assessed as time until CT more than 6 days in early treatment group and all patients in late treatment group (*p* < 0.05). However, %GGO, % consolidation, CT disease severity score or real time until CT, viral clearance after treatment, duration of fever after treatment, or time until hospital discharge demonstrated significant differences between patients assessed as time until CT equal to or less than 6 days in early treatment group and those assessed as time until CT more than 6 days in early treatment group and all patients in late treatment group (*p* < 0.05).

**Table 5:** Differences between patients whose time until CT was 6 days or less and those whose time until CT was more than 6 days

			TV	SE (%)	SP (%)	PPV (%)	NPV (%)	AC (%)	Real early and late treatment groups (cases)	Viral clearance in the first 6 days (days) [median]	Duration of fever (≥ 37.5 °C or ≥ 37.0 °C) (days) [median]	Time duration until hospital discharge (days) [median]
Radiological prediction	% GGO	6 days or less	8 ≤	73.7 (14/19)	76.9 (10/13)	82.4 (14/17)	66.7 (10/15)	75.0 (24/32)	11	4	4	16
		More than 6 days							21	5	3	23
		*p* value	N/A						N/A	0.13	0.67	0.03
	% Consolidation	6 days or less	< 0.33	94.4 (15/19)	76.9 (10/13)	83.3 (15/18)	71.4 (10/14)	78.1 (25/32)	9	4	1.5	15
		More than 6 days							23	5	3	22.5
		*p* value	N/A						N/A	0.1	0.01	0.03
	Combined method	6 days or less	0.40 <	94.7 (18/19)	61.5 (8/13)	78.3 (18/23)	88.9 (8/9)	81.3 (26/32)	11	4	2	16
		More than 6 days							21	5	3.5	23
		*p* value	N/A						N/A	0.02	0.002	0.02
	CT disease severity score	6 days or less	≤ 5	73.7 (14/19)	84.6 (11/13)	87.5 (14/16)	68.8 (11/16)	78.1 (25/32)	7	3	2.5	15
		More than 6 days							25	5	3.5	23
		*p* value	N/A						N/A	0.04	0.1	0.01
Patients whose time until CT equal to or less than 6 days in early treatment group vs. patients assessed as time until CT more than 6 days in early treatment group and all patients in late treatment group		6 days or less	N/A						9	4	2	15
		More than 6 days							23	5	3	23
		*p* value							N/A	0.01	0.15	0.0004

*TV* threshold value, *SE* sensitivity, *SP* specificity, *PPV* positive predictive value, *NPV* negative predictive value, *AC* accuracy

## Discussion

Our results demonstrated that quantitatively and qualitatively assessed radiological indexes, especially combined as quantitatively assessed indexes, had equal or superior capability for prediction of therapeutic effect by favipiravir treatment in relation to time between onset of clinical symptoms and CT examination (time until CT) in COVID-19 patients with CT enrolled in a previously published multicenter clinical trial [15]. In addition, ML-based CT texture analysis software for assessing radiological findings for COVID-19 patients was found to be equally or more useful than visually assessed CT disease severity and actual time until CT in this setting. To our knowledge, this is the first paper to report the capabilities of quantitatively assessed radiological findings by ML-based CT texture analysis software and disease severity visually assessed on CT and directly compare them with the time until CT for COVID-19 patients treated with favipiravir.

When the relationship between time until CT examination and quantitatively and qualitatively assessed radiological findings was evaluated, % GGO and % consolidation evaluated by the ML-based CT texture analysis software and CT disease severity score had significant negative or positive correlations with time until CT. In addition, % consolidation and % GGO were significant predictors for time until CT based on the results of stepwise regression analysis in this cohort. These findings were compatible with previously published radiological studies for COVID-19 [28–33].

Evaluation of differentiation and patient outcome prediction capabilities for patients whose time until CT was equal to or less than 4, 5, or 6 days in early treatment group and those whose time until CT was more than the respective number of days in early treatment group and all patients in late response group indicated that all quantitatively or qualitatively evaluated CT indexes had the capability to serve as discriminators in this setting. In addition, the accuracy of the combined quantitative index was significantly higher than that of the CT disease severity score for patients whose time until CT was 4 days. Moreover, sensitivities of combined quantitative index and % consolidation were significantly higher than that of % GGO for determination of patients whose time until CT was 5 days. Likewise, viral clearance after treatment, duration of fever after treatment, or time until hospital discharges determined using all radiological indexes and time until CT showed significant differences between COVID-19 patients whose time until CT was equal to or less than 4, 5, or 6 days in early treatment group and those whose time until CT was more than the respective number of days in early treatment group and all patients in late treatment group. Also, the capability of the combined quantitatively assessed index method using ML-based CT texture analysis software, to predict patient outcome, was considered to be equal or superior to that of % GGO, % consolidation, CT disease severity, or real time until CT. Furthermore, the number of patients selected each day by means of the combined quantitative index was more than that determined by time until CT. These findings suggest that there were COVID-19 patients whose quantitative CT findings were milder than what would be indicated by their time until CT, and thus might be considered as good candidates for favipiravir treatment based on the ML-based CT texture analysis results for this cohort. According to the original randomized trial of patients with asymptomatic to mildly symptomatic COVID-19 [14], administration of favipiravir did not significantly improve viral clearance in the first 6 days, but after that viral clearance tended to occur earlier with use of the agent. Favipiravir was also associated with a significant improvement in fever observed the day after starting therapy, compared with findings for no therapy. Our results therefore also imply that favipiravir would be more efficacious if administered to COVID-19 patients who were assessed as less than 1 week from clinical onset on CT. Moreover, our findings suggest that the ML-based CT texture analysis-based quantitative assessments, including the combined quantitative index rather than the CT disease severity score, would be more suitable for a more accurate selection of COVID-19 patients for treatment with favipiravir as compared with using only real time until CT in this setting, although further validation of our results in future studies is warranted.

There are several limitations to this study. First, the study population in this study was small and selected retrospectively from a previously published multicenter clinical trial with a relatively small sample size. In addition, the sample size of the early treatment group determined by time until CT and others was even smaller. Moreover, the original study design assessed safety and therapeutic effect of favipiravir for the early and late treatment groups divided according to original study enrollment, but not based on the time between onset of clinical symptoms and treatment or CT examination. Therefore, only a limited number of patients were treated within the time for evaluation after onset of clinical symptoms and examined by CT before treatment initiation. These circumstances may have affected our study results, so that further evaluation in a randomized trial with a larger sample size is clearly warranted. Second, we used proprietary software based on machine learning for evaluating CT findings in COVID-19 patients. Although this software was based on previously published machine learning software used for various pulmonary parenchyma diseases with proven capability to serve as a second reader to support expert radiologists and improve their intra- and inter-observer agreements of CT evaluation, there have been no reports concerning the effect of this software on agreements for radiological assessments of CT for COVID-19 patients. Moreover, no training, validation, or test case studies of this software have been published at this time. Therefore, further evaluation of the software is also warranted, and we plan for these studies using software improvements based on results of the current study in the near future. Third, this study analyzed all CT data with the same proprietary software provided by Canon Medical Systems, and not with additional software provided by other vendors or developed by other academics [34, 35]. All CT data were obtained from different CT systems from various CT vendors, which use different detector row systems and CT protocols with various automatic exposure control systems, radiation doses, reconstruction algorithms, section thicknesses, etc. These differences may have impacted our study results, especially quantitatively. Fourth, although the results suggested that CT has the capability to identify patients who have been examined within 6 days from onset of clinical symptoms and, therefore, would be good candidates for favipiravir therapy, no direct comparisons were made of favipiravir treatment outcomes for patients with quantitatively and qualitatively assessed radiological indexes on CT and time after onset of clinical symptoms.

In conclusion, ML-based CT texture analysis is equally or more useful for predicting time until CT for favipiravir treatment on COVID-19 patients than CT disease severity score. In addition, ML-based CT texture analysis may have a better potential for predicting the effect of favipiravir treatment on COVID-19 patients than CT disease severity score.
